# Association of Higher Caries Levels With Reduced Oral Health-Related Quality of Life Among Somali School Children in Hargeisa

**DOI:** 10.1016/j.identj.2026.109766

**Published:** 2026-08-11

**Authors:** Abdelrahman Eid Dahroug, Espen Heen, Ibrahimu Mdala, Mohamed Hussein, Ahmed Ali Madar

**Affiliations:** a Department of Community Medicine and Global Health, Institute of Health and Society, Faculty of Medicine, University of Oslo, Oslo, Norway; b Department of Nursing, Health and Laboratory Science, Faculty of Health, Welfare and Organisation, Ostfold University College, Fredrikstad, Norway; c Department of General Practice, Institute of Health and Society, Faculty of Medicine, University of Oslo, Oslo, Norway; d Department of Planning, Policy and Strategic Information, Ministry of Health Development, Hargeisa, Somaliland

**Keywords:** Oral health, OHRQoL assessment, Dental caries, Somali children, Observational study

## Abstract

**Background:**

Evidence on how Somali children are affected by their oral health status is limited. This study aimed to assess oral health-related quality of life (OHRQoL) among 12-year-old schoolchildren in Hargeisa, Somaliland, and to examine the potential association of dental caries status (DMFT index) with OHRQoL.

**Methods:**

A cross-sectional study was conducted in Hargeisa in December 2022, in which 405 children were randomly selected from 16 primary public and nonpublic schools using 2-stage probability cluster sampling. Data collection involved clinical examinations, and OHRQoL was assessed using six items from the WHO Child Oral Health Questionnaire (2013), capturing pain, functional, and psychosocial impacts. Associations were analysed using Poisson regression with robust variance estimation, accounting for clustering at the school level and adjusted for sociodemographic and behavioural factors.

**Results:**

Approximately 71% reported at least one OHRQoL impact, predominantly toothache (54.7%) and eating difficulty (50.1%). Only 24% had a score > 2 impacts (on a 0-6 scale). Compared to public school peers, students from nonpublic schools reported significantly higher levels of dissatisfaction with the appearance of their teeth, thus avoiding smiling and laughing. In multivariable analysis, higher caries levels (DMFT ≥2) were associated with an increased prevalence of OHRQoL impacts (prevalence ratio [PR] = 1.15; 95% CI: 1.01-1.29). Urban residence (PR = 1.15; 95% CI: 1.03-1.28), dental visits in the past 12 months (PR = 1.34; 95% CI: 1.18-1.53), and poor self-rated oral health (PR = 1.40; 95% CI: 1.24-1.58) were also associated with higher prevalence of impacts.

**Conclusion:**

Despite relatively low mean caries levels among 12-year-old school children in Hargeisa, higher caries levels (DMFT ≥2) were associated with an increased prevalence of OHRQoL impacts. The promotion of oral health education alongside preventive dental health programs specifically designed for Somali school children is recommended.

## Introduction

Oral health is integral to overall health, well-being and quality of life, enabling an individual to achieve full capacity and participation in society from birth to old age.^1^^,^^2^ Tooth loss is mainly the ultimate outcome of a lifelong experience of severe dental caries and periodontal disease. It greatly affects the quality of life, reducing functional capacity (eg, chewing and speech), appearance, self-esteem, and social relationships.^3^

The dimensions of oral health-related quality of life (OHRQoL) encompass various aspects, including pain, functional abilities, emotional and psychological factors, as well as social impacts. These effects can vary in intensity, ranging from life-threatening conditions like oral cancers to chronic issues such as caries and periodontitis, acute problems like toothaches and oral mucosal lesions, and aesthetic concerns like malocclusion.^4^ Currently, the most common chronic disease among children is dental caries with a prevalence seven times more common than seasonal allergies and five times more common than asthma.^5^ Discomfort, pain, disfigurement, acute and chronic infections, eating and sleep disruption in addition to high treatment costs, higher risk of hospitalization, and loss of school days, are different forms that illustrate how severe caries can detract from children’s quality of life.^1^

WHO emphasizes the need to monitor oral health through metrics that capture population experiences of poor OHRQoL and the availability, accessibility, and responsiveness of primary oral healthcare systems. Key indicators include monitoring reductions in the proportion of children (ages 5, 6, and 12) and adolescents (age 15) reporting OHRQoL reduction, as well as improvements in their access to essential oral health services.^3^ Key factors influencing OHRQoL among child populations in Africa could include individual factors (eg, demographic, personal biological and psychological status), behavioural factors (eg, poor dietary habits and irregular brushing), environmental factors (eg, socio-economic status, education, type of residence and utilization of dental services), and the presence of any oral condition other than dental caries.^4^ Based on this framework, it is hypothesized that increasing levels of dental caries impair children’s OHRQoL through multiple pathways, including pain and discomfort, functional limitations (eg, difficulty eating), and psychosocial impacts (eg, reduced self-esteem and social interaction), which are potentially modified by individual, behavioural, and socioeconomic factors.

It is highly recommended to prioritize interventions targeting young age. Adverse social conditions and events in early life can negatively impact health later in adult life, and even across subsequent generations.^6^ Information on the OHRQoL of this population could emphasize developing oral health promotion and care programmes.^7^

Somaliland has a population of roughly 4.5 million; individuals aged 24 or younger make up nearly half of the population, and females comprise 52%.^8^ Given the limited availability of population-based data on oral health and OHRQoL in this setting, the present study is part of a school-based survey conducted in Hargeisa, a city populated by over one million residents. Earlier findings from this survey showed that the overall prevalence of dental caries was 62.7%, and the mean DMFT (Decayed, Missing, Filled Teeth) was found to be 1.7 among 12-year-old schoolchildren.^9^ The objective of the study was to assess OHRQoL among 12-year-old schoolchildren, and to investigate the association between dental caries status (DMFT index) and OHRQoL while adjusting for relevant associated factors.

## Methods

This cross-sectional study was carried out in December 2022 in Hargeisa district of Maroodijieh region, which hosts the largest concentration of primary schools nationwide. Demographically, boys accounted for 56.1% of total enrolment across grades 1 to 8, while nearly three-fifths (58%) of primary students attended public schools. Rural areas constituted just 10% of total primary school enrolment.^10^

### Sampling procedure

#### Sample size

The sample size for this baseline oral health survey was primarily calculated based on dental caries prevalence to ensure adequate precision in estimating the population burden. In addition, the sample size was considered sufficient to detect a meaningful difference in OHRQoL outcomes between exposure groups, consistent with similar studies in comparable settings.^7^^,^^11^

A sample size of 405 schoolchildren was determined based on the following parameters: a 50% prevalence of a DMFT score ≥1 (derived from recent studies in comparable contexts),^12^ a minimum clinically relevant effect size of 33%, a 2:1 ratio of public to private school enrolment, a precision margin of 5%, 80% statistical power, a design effect of 1.2, and an anticipated 5% nonresponse rate. The chosen design effect was guided by earlier evidence in Somaliland that demonstrated low clustering effects and minor variations between socioeconomic status groups.^13^^,^^14^

#### Two-stage cluster sampling

##### Selection of schools

The sampling frame was derived from the Ministry of Education’s list of primary schools in Hargeisa district. From the 334 schools listed, 16 were randomly chosen without initial stratification. The final selection included nine non-public and seven public primary schools, with two of the public schools located in rural regions.

##### Selection of participants

**Fig. 1:**
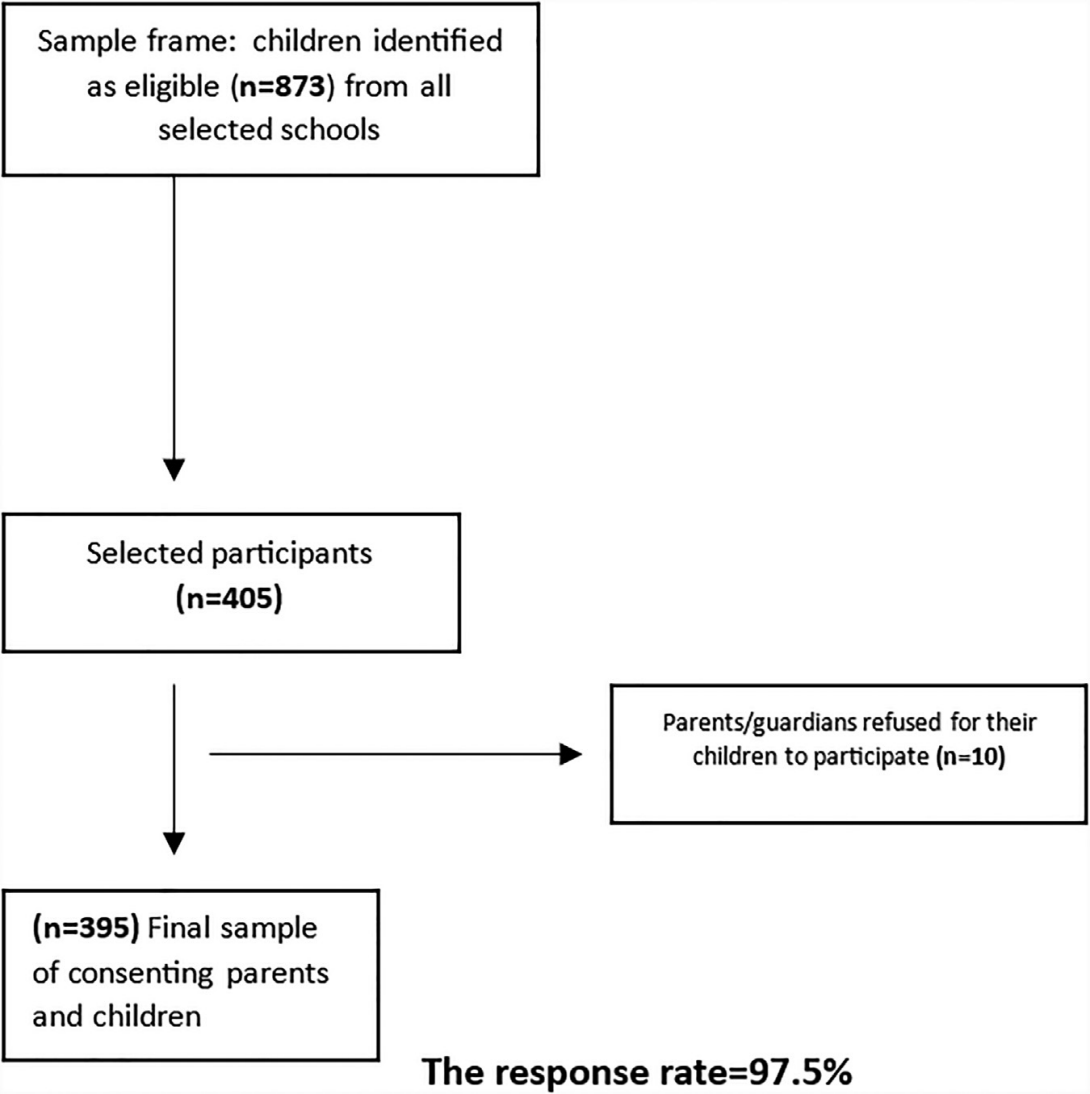
Flow chart exemplifying steps towards achieving a final sample.

A total of 873 students from the selected schools fulfilled the eligibility criteria, with 405 participants randomly selected from this pool for the study (Figure 1). Eligible participants were exclusively 12-year-olds (born in 2010) residing in Hargeisa district, with no severe congenital or acquired health conditions, and who submitted informed consent forms signed or thumb-imprinted by parents or guardians. The research team collected these consent documents directly from students’ classrooms on the subsequent school day.

### Data collection

A combination of oral clinical examination and self-assessment of oral health was employed using the WHO structured questionnaire for children from the *Oral Health Surveys: Basic Methods* (5th ed., 2013, pp. 115-118). Both assessments were conducted on school premises. Seven locally licensed dentists were hired as research assistants, divided into two teams: three examiners trained for clinical assessments and four interviewers for administering questionnaires.

Clinical examinations were carried out under natural daylight and included calculating DMFT scores and evaluating the necessity for dental treatment. Dental caries was recorded at the cavitation level (D3 threshold), in accordance with WHO criteria for oral health surveys. A tooth was recorded as decayed when a lesion in a pit or fissure, or on a smooth surface, presented with an unmistakable cavity, undermined enamel, or a softened floor or wall. All erupted permanent teeth, excluding third molars, were examined and included in the DMFT index. Teeth were classified as decayed (D), missing due to caries (M), or filled (F). The DMFT score was calculated as the sum of these components for each individual.^3^

The questionnaire was first translated from English to Somali by a Somali dentist, then independently back-translated into English by a separate interpreter to validate accuracy. It gathered data on parental education levels, self-rated dental status, frequency of dental visits, oral hygiene practices (eg, tooth-cleaning frequency), and OHRQoL impacts over the past year.^3^ Interviewers recorded participants’ responses directly into the questionnaire, with data later transferred to digital spreadsheets. Detailed question wording, response options, and coding schemes are provided in the Supplementary File.

### Pilot study

A pilot study, which involved 26 pupils from a non-public school, was conducted to assess the accuracy and reliability of the Somali-translated WHO questionnaire, reduce potential inconsistencies among interviewers, and standardize procedures for the clinical examination team. Although formal kappa statistics were not calculated, efforts were made to standardize the examination procedures through training and supervision. The training involved differentiating between retained deciduous teeth and permanent teeth and identifying decayed, filled, missing teeth due to caries, and unerupted teeth, and accordingly noting down numbers (for permanent teeth) and letters (for deciduous teeth) precisely in the assessment sheet, in addition to evaluating the level of intervention needed for each participant. The examination was performed in three sessions (8-9 pupils per session) on the same day, under the supervision of the main researcher (an experienced dentist). At the end of each session, findings were reviewed collectively to ensure consistency in identifying dental conditions and recording data. Following the pilot, the research team held debriefing session to confirm that the translated questions retained their original meaning and intent. The principal researcher also supervised the clinical assessments during the main survey. Detailed methodologies, including measurement protocols and pilot study, are provided in the Supplementary Materials. The pilot findings indicated that no substantial modifications to the questionnaire or measurement protocols were necessary, confirming their adequacy for the main study.

### Data protection and statistical analysis

Collected data were securely stored using the Sensitive Data Service (TSD) provided by the University of Oslo (UiO). Data entry and statistical analysis were performed in STATA version 18. Caries prevalence was defined as the presence of untreated decayed teeth (DT) in either permanent or primary teeth, while the D component referred specifically to untreated decay in permanent teeth only. Although DMFT is a cumulative measure, in this study it was overwhelmingly driven by the decayed component, indicating that it largely reflects untreated caries and current disease burden. To distinguish between children with no or minimal caries experience and those with a higher disease burden, DMFT was categorized into two groups (<2 and ≥2). This categorization was informed by the distribution of the data, which showed a relatively high prevalence of caries but low overall severity (mean DMFT = 1.7), and was applied to enhance interpretability and ensure stable regression estimates.

To streamline the presentation of results in Table 2, several variables were recategorized during analysis. Parents’ education levels were collapsed into four tiers: *low* (no formal schooling or primary education), *medium* (secondary education), *high* (university education), and *unknown* as a separate category for participants who reported (don’t know). Teeth-cleaning frequency was dichotomized into *regular* (once or more daily) and *irregular* (weekly or monthly) due to sparse data in the weekly and monthly categories. Self-rated dental health was grouped as *good* (excellent, very good, good, or average) vs *poor* (poor or very poor). For events occurring in the past 12 months: (1) Dental visits were classified as *yes* (at least once) or *no*; (2) Toothache/discomfort frequency was categorized as *yes* (often, occasionally, rarely) or *no*; and (3) Other OHRQoL items – including dissatisfaction with dental appearance, avoiding smiling/laughing due to teeth, teasing by peers, school absenteeism linked to dental issues, and eating difficulties – were dichotomized into *yes* or *no.*

The prevalence of each OHRQoL item was first assessed individually. Then, a composite scale was made to measure the intensity of reduced OHRQoL per participant, ranging from 0 (optimal – all responses ‘no’) to 6 (worst condition – all responses ‘yes’). Differences in proportions for each variable across OHRQoL items were assessed using Pearson’s chi-square test. Due to the skewed distribution of DMFT scores, the Mann–Whitney *U* test was used to compare the rank sum between the groups.

**Fig. 2:**
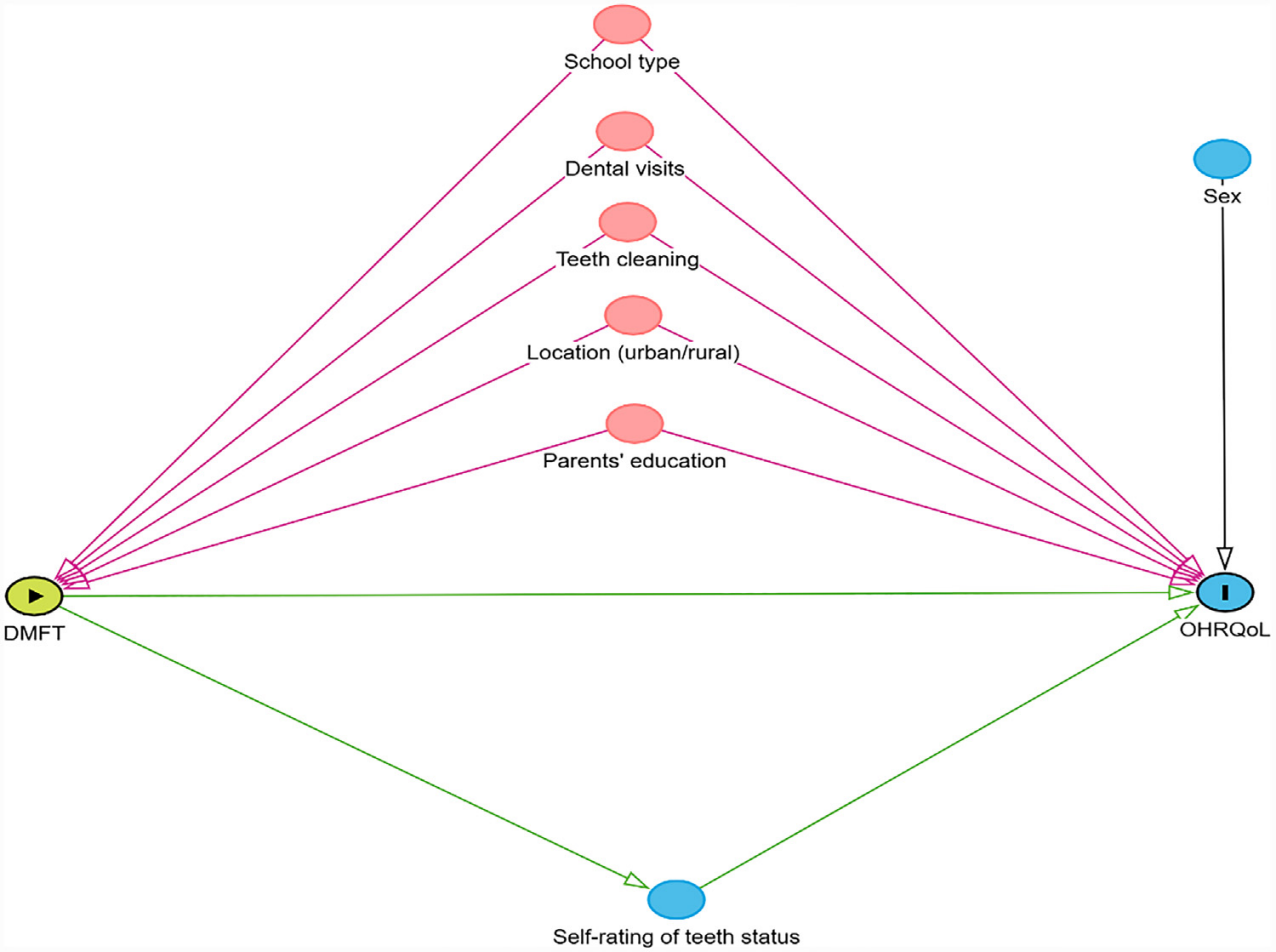
Directed acyclic graph (DAG) illustrating the hypothesized relationships between dental caries status (DMFT), related variables, and the OHRQoL.

Interaction analysis confirmed no sex-based variation in the effect of DMFT (exposure) between boys and girls (*P* value = .86), and the calculated intraclass correlation coefficient was 0.029. Given the high prevalence of the outcome and the clustered sampling design, Poisson regression models with robust variance estimation were used to assess the association between DMFT and OHRQoL impacts. Based on prior literature and conceptual relevance, self-rated teeth status as a potential mediator and other potential confounders, including school type, location, parents’ education, tooth-cleaning frequency, and dental visits, were controlled for in the multivariable model (Figure 2). Data collected from the pilot study were not included in the final analysis.

### Ethical consideration

This study received ethical approval from the Norwegian Regional Committees for Medical and Health Research Ethics (REK South-East; Reference: 2022/494598) and the Norwegian Centre for Research Data. Ethical clearance was also secured from Somaliland’s Ministry of Health Development (MOHD; Reference: MOHD/VM:3/509/2022), alongside permissions from the Ministry of Education and participating schools. Eligible participants were provided with Somali-language consent forms requiring parental/guardian signatures in case they accepted their children to participate in the study. Following clinical examinations, children received personalized oral health feedback, hygiene instructions, and toothpaste/toothbrushes. Parents were notified if urgent dental care was needed. For the pilot study, prior approval from school administration and verbal consent from participants were secured. In accordance with the global code of conduct for research in resource-poor settings, local research assistants got the knowledge and experience required for conducting an oral health survey. Upon the study’s completion, the findings were formally communicated to local authorities.^15^

## Results

A total of 395 schoolchildren from 16 schools participated in the survey, of whom six were absent during the dental clinical examination. Only 16% of participants were from rural areas and belonged to public schools. Despite a third of the children providing ‘don’t know’ responses when asked about their parents’ educational background, significant differences between school types were observed (Table 1).

**Table 1:** Socio-demographic characteristics and clinical indicators of public and non-public school attendees.

Variable	Total (*N* = 395) (%)	Public schools (*N* = 232) (%)	Nonpublic (*N* = 163) (%)	*P* value
**Sex**				
Boys	229 (58.0)	128 (55.2)	101 (62.0)	.18
Girls	166 (42.0)	104 (44.8)	62 (38.0)	
**Location**				
Urban	332 (84.0)	169 (72.8)	163 (100)	<.01*
Rural	63 (16.0)	63 (27.2)	0 (0)	
**Father’s education**				
No formal schooling	70 (17.7)	57 (24.6)	13 (8.0)	<.01*
Primary education or below	60 (15.2)	43 (18.5)	17 (10.4)	
Secondary education	46 (11.7)	20 (8.6)	26 (16.0)	
University education	96 (24.3)	32 (13.8)	64 (39.3)	
Unknown	123 (31.1)	80 (65.0)	43 (35.0)	
**Mother’s education**				
No formal schooling	124 (31.4)	88 (37.9)	36 (22.1)	<.01*
Primary education or below	66 (16.7)	39 (16.8)	27 (16.6)	
Secondary education	41 (10.4)	23 (9.9)	18 (11.0)	
University education	47 (11.9)	14 (6.0)	33 (20.3)	
Unknown	117 (29.6)	68 (58.1)	49 (41.9)	
**Self-reported dental pain (past 12 mo)**				
Often	50 (12.7)	32 (13.8)	18 (11.0)	.82
Occasionally	75 (19.0)	42 (18.1)	33 (20.2)	
Rarely	91 (23.0)	52 (22.4)	39 (23.9)	
Never	179 (45.3)	106 (45.7)	73 (44.8)	
**Oral clinical examination**†				
**Caries prevalence**	244 (62.7)	135 (58.6)	109 (68.5)	.04*
**DMFT mean (±SD)**	1.7 (±1.8)	1.5 (±1.8)	1.9 (±1.8)	.02*
**DT (*n*) (%)**	240 (61.7%)	135 (58)	109 (66)	.06
**FT (*n*) (%)**	3 (0.7)	1 (0.4)	2 (1.2)	.46
**MT (*n*) (%)**	4 (1)	3 (1.3)	1 (0.6)	.55

*N* = 395. Parent’s education: responses of ‘Unknown’ were a separate category, not representing missing data. Caries prevalence: untreated caries in either permanent or primary teeth.

⁎ Bold values indicate statistically significant differences (p < 0.05), [Pearson’s chi-square test for the proportions and two-sample Wilcoxon rank-sum (Mann–Whitney) test for DMFT given its skewed distribution].

† Clinical parameters (DMFT and Caries): (*n* = 389) (as six students were absent during the dental clinical examination).

Among the schoolchildren, 71.1% indicated experiencing one or more of the six OHRQoL items assessed. The highest impacts were reported on dental pain, eating difficulty, and missing classes due to toothache or discomfort. A quarter (total 25.3%) of the students reported social impairments in terms of dissatisfaction with the appearance of teeth, being teased about their teeth, or avoiding smiling (Table 2).

**Table 2:** Frequency of OHRQoL items ‘assessed using a 12-month recall period’ across socio-demographics, behavioural- and clinical dental caries indicators.

Variables	Total (*N* = 395) (%)	Toothache/discomfort (*N* = 395) (%)	Missing class or school day (*N* = 395) (%)	Eating difficulty (*N* = 395) (%)	Appearance dissatisfaction (*N* = 395) (%)	Avoiding smiling (*N* = 395) (%)	Others make fun (*N* = 395) (%)
Frequency of each OHRQoL item† (*N* = 395) (%)		216 (54.7)	71 (18.0)	198 (50.1)	55 (13.9)	49 (12.4)	40 (10.1)
Sex *Boys*	229 (58.0)	132 (57.6)	34 (14.8)	105 (45.8)	36 (15.7)	26 (11.3)	25 (10.9)
*Girls*	166 (42.0)	84 (50.6)	37 (22.3)	93 (56)	19 (11.5)	23 (13.9)	15 (9.0)
		*P = .16*	*P = .06*	P = .04*	*P = .23*	*P = .46*	*P = .54*
School type *Public*	232 (58.7)	126 (54.3)	40 (17.2)	121 (52.2)	17 (7.3)	22 (9.5)	19 (8.2)
*Non-public*	163 (41.3)	90 (55.2)	31 (19.0)	77 (47.2)	38 (23.3)	27 (16.6)	21 (12.9)
		*P = .86*	*P = .65*	*P = .34*	<.01*	P = .04*	*P = .13*
Location *Urban*	332 (84.0)	177 (53.3)	63 (19.0)	176 (53.0)	55 (16.6)	43 (13.0)	39 (11.8)
*Rural*	63 (16.0)	39 (61.9)	8 (12.7)	22 (34.9)	0 (0)	6 (9.5)	1 (1.6)
		*P = .21*	*P = .23*	P = .01*	<.01*	*P = .45*	P = .01*
Father’s education *Low*	130 (32.9)	76 (58.5)	21 (16.1)	67 (51.5)	13 (10.0)	17 (13.1)	13 (10.0)
*Moderate*	46 (11.7)	28 (60.9)	13 (28.3)	29 (63.0)	7 (15.2)	6 (13.0)	3 (6.5)
*High*	96 (24.3)	50 (52.1)	22 (22.9)	44 (45.8)	21 (21.9)	16 (16.7)	11 (11.5)
Unknown	123 (31.1)	62 (50.4)	15 (12.2)	58 (47.2)	14 (11.4)	10 (8.3)	13 (10.6)
		*P = .45*	P = .04*	*P = .23*	*P = .06*	*P = .29*	*P = .83*
Mother’s education *Low*	190 (48.1)	112 (59.0)	41 (21.6)	95 (50.0)	26 (13.7)	26 (13.7)	19 (10.0)
*Moderate*	41 (10.4)	23 (56.1)	8 (19.5)	27 (65.8)	6 (14.6)	4 (9.8)	5 (12.2)
*High*	47 (11.9)	24 (51.1)	8 (17.0)	22 (46.8)	10 (21.3)	8 (17.0)	7 (14.9)
Unknown	117 (29.6)	57 (48.7)	14 (12.0)	54 (46.2)	13 (11.1)	11 (9.4)	9 (7.7)
		*P = .34*	*P = .20*	*P = .17*	*P = .41*	*P = .49*	*P = .55*
Teeth cleaning *Regular (daily)*	365 (90.1)	188 (52.8)	66 (18.5)	171 (48.0)	52 (14.6)	47 (13.2)	39 (11.0)
*Irregular (weekly/monthly)*	39 (9.9)	28 (71.8)	5 (12.8)	27 (69.2)	3 (7.7)	2 (5.1)	1 (2.6)
		P = .02*	*P = .37*	***P = .01***	*P = .24*	*P = .15*	*P = .09*
Dentist’s visits *No*	337 (85.3)	166 (49.3)	49 (14.5)	161 (47.8)	45 (13.4)	40 (11.9)	29 (8.6)
*Yes*	58 (14.7)	50 (86.2)	22 (37.9)	37 (63.8)	10 (17.2)	9 (15.5)	11 (19.0)
		<.01*	<.01*	P = .02*	*P = .42*	*P = .44*	P = .02*
Self-rating of teeth status *Good*	290 (73.4)	130 (44.8)	38 (13.1)	114 (39.3)	44 (15.2)	41 (14.1)	31 (10.7)
Poor	105 (26.6)	86 (81.9)	33 (31.4)	84 (80.0)	11 (10.5)	8 (7.6)	9 (8.6)
		<.01*	<.01*	<.01*	*P = .23*	*P = .08*	*P = .54*
Clinical indicators‡ DMFT *mean (±SD)*	1.7 (±1.8)	1.9 (±1.9) (**If no**) 1.3 (±1.6)	2.3 (±2) (**No**) 1.5 (±1.7)	1.9 (±1.8) (**No**) 1.4 (±1.7)	1.8 (±1.9) (**No**) 1.6 (±1.8)	1.6 (±1.7) (**No**) 1.7 (±1.8)	1.6 (±1.6) (**No**) 1.7 (±1.8)
		<.01*	<.01*	*P = .02*	*P = .61*	*P = .87*	*P = .92*
DMFT = 0	149 (38.3)	70 (47.0)	22 (14.8)	71 (47.7)	18 (12.1)	17 (11.4)	14 (9.4)
DMFT >0	240 (61.7)	143 (59.6)	48 (20.0)	124 (51.7)	35 (14.6)	31 (12.9)	24 (10.0)
		P = .01*	*P = .19*	*P = .44*	*P = .48*	*P = .66*	*P = .84*
Untreated caries§ *Yes*	244 (62.7)	142 (58.2)	50 (20.5)	127 (52.1)	37 (15.2)	34 (13.9)	24 (9.8)
*No*	145 (37.3)	71 (49.0)	20 (13.8)	68 (46.9)	16 (11.0)	14 (9.67)	14 (9.7)
		*P = .20*	*P = .25*	*P = .62*	*P = .20*	*P = .44*	*P = .16*

*N* = 395. (**if no**): category/group of participants who did not report experiencing this OHRQoL item.

⁎ P-values are presented in italics; statistically significant values (p < 0.05) are shown in bold italics, [Pearson’s chi-square test for the proportions and two-sample Wilcoxon rank-sum (Mann–Whitney) test for DMFT given its skewed distribution].

† Responses are not mutually exclusive.

‡ Clinical parameters (DMFT and caries): total sample (*n* = 389) only.

§ Untreated caries (either in permanent or deciduous teeth).

The intensity scale (impacts per participant) showed that the overall average (SD) was 1.6 (±1.4). Among students, 28.9% had optimum condition with a score of zero, 24% had a score > 2, and only 10.1% had a score >3. Furthermore, 78.8% of those with eating difficulty reported dental pain (*P* value <.01).

The bivariate analysis of the background variables with OHRQoL showed significant differences. Participants from non-public schools were significantly more dissatisfied with the appearance of teeth, avoiding smiling and laughing, than their peers from public schools (Table 2).

Compared to rural areas, appearance dissatisfaction, others making fun, and eating difficulty were higher among students in urban areas. Eating difficulty and dental pain were higher among those with irregular teeth cleaning. Eating difficulty, dental pain, and missing classes due to toothache were higher among those with dental visits during the past 12 months and those who rated their teeth status as poor (Table 2).

As we previously reported, the overall mean DMFT (SD) was 1.7 (±1.8) and 45.8% of participants had DMFT ≥2 scores. The majority of the DMFT index was attributed to the ‘DT’ component, with only 1% of participants having missing permanent teeth due to caries, and 0.8% receiving dental fillings. Only 14.7% of the participants had dental visits in the past year.^9^ Students with eating difficulty, pain, and missing classes had a significantly higher mean DMFT (Table 2).

Students with impacts on OHRQoL (score> zero) had relatively higher mean DMFT with 1.8 (±1.8), compared to 1.3 (±1.6) for absence of these impacts (*P* value <.01). In addition, they had much more dental visits (80.4% vs 19.6%, *P* value <.01). Students, who rated their teeth status as poor, had a significantly higher mean DMFT with 2.1 (±2) (*P* value <.01), compared to 1.5 (±1.6) for good status. In addition, they had more dental visits (22.9% for poor vs 11.7% for good status) (*P* value <.01).

In the multivariable analysis, higher dental caries levels were significantly associated with OHRQoL impact. Children in DMFT ≥2 category had a 15% higher prevalence of reporting at least one impact (Table 3). Urban residence, recent dental visits, and poor self-rated oral health were also associated with higher prevalence of impacts. In sensitivity analysis, exclusion of self-rated teeth status from the adjusted model resulted in a slightly higher estimate (prevalence ratio = 1.18; 95% CI: 1.02-1.36), compared to the fully adjusted model (prevalence ratio = 1.15; 95% CI: 1.01-1.29), suggesting that it may partially influence the relationship. No significant associations were found for sex, school type, or parents’ education levels.

**Table 3:** Association between dental caries status (DMFT) and OHRQoL impacts among schoolchildren using Poisson regression with cluster-robust variance (school level), with prevalence ratios (PRs) and 95% confidence interval (CI).

Explanatory variables	Unadjusted		Adjusted*	
	PR (95% CI)	*P* value	PR (95% CI)	*P* value
DMFT (ref: 0)				
1	0.95 (0.76, 1.19)	.67	0.96 (0.78, 1.16)	.69
≥2	1.19 (**1.04, 1.37**)	**.01**	1.15 (**1.01, 1.29**)	**.03**
Sex (ref: Boy)				
Girl	1.02 (0.88, 1.20)	.35	1.00 (0.88, 1.11)	.97
School type (ref: Public)				
Non-public	0.97 (0.83, 1.14)	.72	0.88 (0.73, 1.03)	.11
Location (ref: Rural)				
Urban	1.11 (**1.002, 1.23**)	**.046**	1.15 (**1.03, 1.28**)	**.01**
Father’s education (ref: Low)				
Moderate	1.06 (0.87, 1.29)	.53	1.08 (0.88, 1.32)	.42
High	0.99 (0.83, 1.17)	.91	1.01 (0.79, 1.23)	.93
Unknown	0.96 (0.82, 1.13)	.67	1.05 (0.91, 1.20)	.49
Mother’s education (ref: Low)				
Moderate	1.01 (0.82, 1.23)	.94	1.00 (0.79, 1.27)	.97
High	0.99 (0.81, 1.21)	.96	1.04 (0.80, 1.36)	.72
Unknown	0.92 (0.79, 1.08)	.35	0.94 (0.84, 1.05)	.32
Teeth cleaning (ref: Regular [daily])				
Irregular (weekly/monthly)	1.09 (0.97, 1.22)	.13	0.88 (0.75, 1.02)	.89
Dental visits last 12 mo (Ref: No visits)				
Once or more	1.41 (**1.30, 1.53**)	**<.01**	1.34 (**1.18, 1.53**)	**<.01**
Self-rating of teeth status (ref: Good)				
Poor	1.45 (**1.29, 1.63**)	**<.01**	1.40 (**1.24, 1.58**)	<**.01**

*N* = 389. Significant association (the bold values in table).

⁎ Adjusted for sex, school type, location, parents’ education, teeth cleaning, and dental visits, self-rating of teeth status.

## Discussion

This study found that 12-year-old schoolchildren in Hargeisa commonly experienced impacts on their OHRQoL, mainly toothache and eating difficulty. Around 24% of the students had a score >2 (out of 6). Participants from non-public schools were more dissatisfied with the appearance of teeth, avoiding smiling and laughing, than their peers from public schools. DMFT ≥2 was positively associated with increased OHRQoL impacts, with no significant differences observed between sexes. Urban residence, dental visits, and poor self-rated oral health were also significantly associated with higher impact prevalence.

The study revealed a widespread reduction in OHRQoL, particularly toothache and eating difficulties, in school children aged 12. These findings are consistent with similar studies on OHRQoL,^16^, ^17^, ^18^, ^19^, ^20^, ^21^ including studies within the African contexts. For instance, investigations in Sudan and Nigeria – using the Child-OIDP (Oral Impacts on Daily Performance) inventory – reported common impacts on OHRQoL (54.6% and 56.5%, respectively) with moderate average intensity. Both studies identified positive associations between untreated dental caries and these impacts, while also noting no notable sex-based disparities in the observed effects.^7^^,^^22^

In our study, the association of self-rating dental health with overall impacts on OHRQoL was much stronger compared to associations with higher DMFT scores, reflecting the subjective nature of these evaluations. Children may report a decline in their oral health when they believe they require treatment, a viewpoint influenced by societal expectations.^4^ Almost three-quarters (73.8%) of Sudanese school children were dissatisfied with their oral health, despite the low prevalence and severity of caries (24%).^7^ Furthermore, self-perceived oral health partially mediated the association between the higher DMFT and impacts on OHRQoL, indicating that greater dental awareness and psychological support may be crucial for improving OHRQoL beyond just treating dental conditions.

Socio-economic factors appear to play a significant role in shaping oral health outcomes and expectations. In a study by Koposova et al, OHRQoL was evaluated among 12-year-olds in two contrasting areas of the Barents region: Arkhangelsk (North-West Russia) and Tromsø (Northern Norway). While dental caries initially showed an independent effect on OHRQoL, this association diminished when controlling for variables such as country of residence, family income, and parental education. Aesthetic concerns and socio-economic factors emerged as stronger predictors of oral health quality, likely reflecting the stark disparities in living standards between Northern Norway and North-West Russia.^23^ In the current study, Urban residence was also associated with increased OHRQoL impact. Compared to public school peers, students from non-public schools reported significantly higher levels of dissatisfaction with the appearance of their teeth, thus avoiding smiling and laughing. This trend may stem from their heightened awareness of oral health care possibilities, suggesting that increased knowledge drives higher expectations and self-reported impacts.

Research on dental malocclusion consistently demonstrates a significant link to oral health-related challenges, with the most pronounced effects observed in social well-being.^24^ Malocclusion can lead to aesthetic and functional problems, often resulting in a perceived need for treatment. The appearance of teeth, particularly affected in malocclusion cases, holds significant importance in children’s lives, especially as they approach puberty.^4^ Based on the observations from the clinical examination in this study, it is strongly advised to prioritize individuals with developing oral abnormalities, especially dental malocclusion that can be modified by simple dental appliances, when determining the urgency of intervention. Strategies for managing caries, caring for primary teeth, ensuring proper eruption of permanent teeth, promoting good oral hygiene, and addressing detrimental oral habits like thumb sucking and mouth breathing are crucial elements in preventing malocclusion at a young age.^25^

Interestingly, the findings revealed notable positive associations between dental visits and reduced OHRQoL. The ‘DT’ component accounted for the bulk of the DMFT index, with only 0.8% of participants having dental fillings and 1% experiencing permanent tooth loss due to caries. This pattern suggests that dental visits predominantly addressed immediate symptom management – such as prescribing pain-relieving medications – rather than offering restorative interventions or preventive care.^9^

Research among junior high school students in China demonstrated that adherence to a healthy dietary pattern was associated with improved periodontal health.^26^ Similarly, a study conducted in disadvantaged schools in North-Eastern Hungary reported that poor oral hygiene practices and unfavourable lifestyle behaviours were associated with worse oral health outcomes.^27^ These findings collectively emphasize the influence of behavioural and socioeconomic factors on children’s oral health in underserved populations, where unmotivated family background with low income, lack of dental education, and insufficient health care services altogether play an important role.

By the age of 12, all permanent teeth, except for the wisdom teeth, should have erupted.^3^ However, in this survey, a significant portion of the participants (57%) still had retained deciduous teeth or unerupted permanent teeth. This can be attributed to delayed tooth eruption (DTE), which can occur due to various factors related to local or systemic conditions.^28^ The overall prevalence of stunting, wasting, and underweight among Somali preschool children varied from medium to high.^29^, ^30^, ^31^, ^32^ In 2020, 21% of children underfive were stunted, 13% were wasted and 14% were underweight in Somaliland.^33^ A study conducted by Reis et al^34^ on a group of Brazilian primary school children found that underweight children have a higher risk of DTE in their permanent dentition, indicating the need for further research on the relationship between nutritional status and its potential impact on DTE.

This study offers valuable baseline insights into the determinants of dental caries and their associated effects on OHRQoL among 12-year-old Somali children enrolled in public and non-public primary schools in Hargeisa. Data were collected using standardized tools, including the WHO questionnaire and clinical assessments performed by qualified dental practitioners. Twelve-year-old children were selected in accordance with WHO recommendations for global oral health monitoring, as this age represents a key stage in the permanent dentition. However, some children at this age may still present with mixed dentition due to delayed eruption or retained primary teeth, which may influence DMFT estimates by potentially underestimating or overestimating caries experience.

Because of limitations in the accuracy of available school registry data (such as inactive schools, lack of birth records, and misclassification of urban/rural status), probability proportional to size sampling was not feasible. Consequently, the sample may not fully represent the underlying student population with respect to school size and type. Selection bias might have been introduced when few students in a non-public school, whose parents reside abroad (diaspora) or in other cities, were excluded due to difficulties in obtaining the required written consent for participation in the survey. The primary public schools in Hargeisa follow a coeducational system with both boys and girls, while many non-public schools adopt a gender-segregated approach. As a result, this study had a relatively smaller sample size of girls from non-public schools.

Although the WHO questionnaire measures self-rated oral health through a single-item question and aspects of OHRQoL through symptom-based questions, it does not fully capture psychosocial, functional, and emotional aspects of oral health. Additionally, apart from self-reported toothache, it lacks multi-item scoring system to capture the intensity or severity of the impacts. Other limitations are the cross-sectional study design, potential recall bias (12-month recall), and challenges in accurately assessing socioeconomic status, particularly parental education (27.5% responded with ‘don’t know’) and income levels, which have been detailed in a previous publication.^9^ Finally, although the WHO questionnaire was translated into Somali and evaluated through a pilot study to ensure clarity, cultural relevance, and consistency, formal psychometric validation of the translated instrument was not conducted. In addition, inter- and intraexaminer reliability were not formally assessed using kappa statistics, which may limit the assessment of measurement reproducibility.

Strategies for addressing dental caries should expand beyond merely lowering DMFT scores to specifically target untreated DT. In light of the findings, Somaliland could establish a public health target ensuring that children aged 12 have no more than untreated decayed tooth and retain all their first molars. To refine these goals, long-term studies and time-series data are essential. Such research would enable accurate identification and validation of risk groups based on varying oral disease severity, ultimately supporting the development of targeted, evidence-based oral health objectives.

Various recommendations aimed at enhancing oral health outcomes in low-income countries should be considered encompassing implementing stricter regulations to limit the marketing of products that contain free sugars, granting tax exemptions for oral care products, fluoridating drinking water (the recommended level of 0.7 milligrams of fluoride per litre),^35^ utilizing mass media platforms such as television or radio broadcasts for oral health education, and integrating oral health promotion and disease prevention into school curricula.^35^

## Conclusion

This study found prevalent impacts on OHRQoL among children aged 12 in Hargeisa, particularly toothache and eating difficulties, with only 24% reporting scores >2. Approximately one-quarter of participants reported social impairments, including dissatisfaction with the appearance of their teeth, being teased about their teeth, or avoiding smiling. This was mainly observed among non-public school attendees. Elevated DMFT scores correlated strongly with eating difficulties, pain, and school absenteeism. In adjusted analysis, higher caries levels (DMFT ≥2) and urban residence were positively associated with increased OHRQoL impacts. Dental visits and poor self-rated oral health were also significantly associated with higher impact prevalence. Recommendations include setting a national target for 12-year-olds, alongside tailored oral health education and preventive programs.

## Conflict of interest

The authors declare that they have no competing interests.

## Appendix Supplementary materials

Image, application 1

## Footnotes

Supplementary material associated with this article can be found in the online version at doi:10.1016/j.identj. 2026.109766.
