# Supplementary material for: Association of Higher Caries Levels With Reduced Oral Health-Related Quality of Life Among Somali School Children in Hargeisa
Source: Int Dent J. 2026 Aug 11;76(5):109766. doi: 10.1016/j.identj.2026.109766 (PMC13487291; doi:10.1016/j.identj.2026.109766)
Supplement: Supplementary file 1 [file mmc1.docx]

**Supplementary file**

**Association of Dental Caries Severity with Impaired Oral Health-Related Quality of Life *Among Somali School Children in Hargeisa***

Abdelrahman Eid Dahroug[ORCID](https://orcid.org/0009-0006-7161-5784)^1^, Espen Kolstad Heen^1,2^, Ibrahimu Mdala^3^, Mohamed A. Hussein^4^ and Ahmed Ali Madar^1^

^1^ Department of Community Medicine and Global Health, Institute of Health and Society, Faculty of Medicine, University of Oslo, Oslo, Norway. ^2^ Department of Nursing, Health and Laboratory Science, Faculty of Health, Welfare and Organisation, Ostfold University College, Halden, Norway. ^3^ Department of General Practice, Institute of Health and Society, Faculty of Medicine, University of Oslo, Oslo, Norway. ^4^ Department of Planning, Policy and Strategic information Ministry of health development, Somaliland.

**Email:** Abdelrahman Eid Dahroug (**Corresponding author**) abdodentalex@gmail.com ; Espen Kolstad Heen -[e.k.heen@medisin.uio.no](mailto:e.k.heen@medisin.uio.no) ; Ibrahimu Mdala [ibrahimu.mdala@medisin.uio.no](mailto:ibrahimu.mdala@medisin.uio.no); Mohamed A. Hussein- [mo.hussein108@gmail.com](mailto:mo.hussein108@gmail.com) ; Ahmed Ali Madar - [a.a.madar@medisin.uio.no](mailto:a.a.madar@medisin.uio.no)

**Contents**

- **Sample size**
- **Measurements and Pilot study**
- **WHO questionnaire (Variables/ response options/ analyzed codes)**

Figure 3. The regions of Somaliland. Hargeisa is the study- city. ^(1)^

**The sample size was calculated with the free software G-power (Universität Düsseldorf: G*Power (hhu.de)).**

**t tests -** Means: Wilcoxon-Mann-Whitney test (two groups)

**Options:**	A.R.E. method

**Analysis:**	A priori: Compute required sample size

**Input:**	Tail(s)	=	Two

Parent distribution	=	Normal

Effect size d	=	0.33

α err prob	=	0.05

Power (1-β err prob)	=	0.8

Allocation ratio N2/N1	=	0.67

**Output:**	Noncentrality parameter δ	=	2.8260526

Critical t	=	1.9678091

Df	=	303.5775

Sample size group 1	=	192

Sample size group 2	=	128

Total sample size	=	320

Actual power	=	0.8043165

Total sample size 320, reaching 405 by considering 1.2 design effect and 5% non-response rate

The project management including personnel and equipment, calibration of examiners, clinical examination, infection control, and arrangement of examination area was adapted from WHO oral health surveys publication-fifth edition.^(2)^

**Measurements**

A- Dental caries index DMFT

Permanent dentition status was recorded using numbered scores (Table 2). A systematic approach to the assessment of the dentition status was adopted, bearing the following points in mind:

• “the examination should proceed in an orderly manner from one tooth or tooth space to the adjacent tooth or tooth space;

• a tooth should be considered present in the mouth when any part of it is visible

• if a permanent and primary tooth occupy the same tooth space, the status of the permanent tooth only should be recorded”.^(2)^

Table 2. Coding the dentition status – primary and permanent teeth^(2)^

“The criteria for diagnosing a tooth status and the coding are as follows:

Code 0 (Sound crown). A crown is coded as sound if it shows no evidence of treated or untreated clinical caries

Code 1 (Carious crown). Caries is recorded as present when a lesion in a pit or fissure, or on a smooth tooth surface, has an unmistakable cavity, undermined enamel, or a detectably softened floor or wall.

Code 2 (Filled crown, with caries). A crown is considered filled, with decay, when it has one or more permanent restorations and one or more areas that are decayed.

Code 3 (Filled crown, with no caries). A crown is considered filled, without caries, when one or more permanent restorations are present and there is no caries anywhere on the crown.

Code 4 (Missing tooth, due to caries). This code is used for permanent or primary teeth that have been extracted because of caries.

Code 5 (Permanent tooth missing due to any other reason). This code is used for permanent teeth deemed to be absent congenitally, or extracted for orthodontic reasons or because of periodontal disease, or trauma.

Code 6 (Fissure sealant). This code is used for teeth in which a fissure sealant has been placed on the occlusal surface, in pits or fissure.

code 7 (Fixed dental prosthesis abutment, special crown or veneer). This code is used under coronal status to indicate that a tooth forms part of a fixed bridge abutment.

Code 8 Unerupted tooth (crown).

Code 9 Not recorded. This code is used for an erupted permanent tooth that cannot be examined for any reason such as orthodontic bands, severe hypoplasia, etc”.^(2)^

For the DMFT index, The D component included all teeth with codes 1 or 2. The M component comprised teeth coded 4 (i.e. missing only due to caries). The F component included teeth only with code 3. ^(2)^

B- Intervention urgency evaluation

The intervention urgency was assessed as follows:

“0 = No treatment needed

1 = Preventive or routine treatment needed

2 = Prompt treatment including scaling (the process of removing the plaque from the surfaces of the teeth) needed

3 = Immediate (urgent) treatment needed due to pain or infection of dental and/or oral origin

4 = Referred for comprehensive evaluation or medical/dental treatment (systemic condition)”.^(2)^

**Pilot study**

A pilot study was conducted to:

- test the accuracy and reliability of the Somali translation of the WHO questionnaires,
- minimize any potential variability among interviewers and establish consistency in the clinical examination team,
- identify the general flow of data collection and quantify time needed for both interview and clinical examination and thus the maximum number of participants per day

This pilot study conducted in a non-public school involved 26 pupils who were examined by each member of the clinical examination team, under the supervision of the main researcher (certified dentist). The calibration process involved differentiating between retained deciduous teeth and permanent teeth and identifying the decayed, filled, missing teeth due to caries, and unerupted teeth and accordingly noting down numbers (for permanent teeth) and letters (for deciduous teeth) precisely in the assessment sheet in addition to evaluating the level of intervention needed for each participant. The examination was performed in three sessions (8-9 pupils per session) on the same day.^(2)^ The pilot results did not lead to any significant modification of the questionnaire or measurement procedures.

**Data collection**

Both the examinations and interviews were carried out on the same day within the school premises. However, in certain cases, they were scheduled on different days to accommodate factors like time availability and the availability of appropriate space within the schools.

Throughout the duration of the clinical examination, personal protective equipment was used. The oral diagnostic instruments were daily sterilized using an autoclave and a high-level disinfectant solution, then dried and packed in sterilization bags.

**Variables from the questionnaire**

| **variables/questions** | **response options** | **analyzed codes** |
| --- | --- | --- |
| **Sex** | Boy  Girl | 1  2 |
| **School type** | Public  Non-public | 1  2 |
| **Location** | Urban  Rural | 1  2 |
| **Dentist`s visit during the past 12 months** | Once  Twice  Three times  Four times  More than four times  I had no visit to dentist during the past 12 months  I have never received dental care/visited a dentist  I don’t know/don’t remember | 1  2  3  4  5  6  7  9 |
| **How often do you clean your teeth?** | Never  Several times a month (2–3 times)  Once a week  Several times a week (2–6 times)  Once a day  2 or more times a day | 1  2  3  4  5  6 |
| **Father`s level of education** | No formal schooling  Less than primary school  Primary school completed  Secondary school completed  High school completed  College/university completed  No male adult in household  Don’t know | 1  2  3  4  5  6  7  9 |
| **Mother`s level of education** | No formal schooling  Less than primary school  Primary school completed  Secondary school completed  High school completed  College/university completed  No male adult in household  Don’t know | 1  2  3  4  5  6  7  9 |
| **self-rating teeth status: How would you describe the health of your teeth and gums?** | excellent,  very good,  good or  average  Poor  very poor  Don’t know | 1  2  3  4  5  6  9 |
| **Frequency of self-assessed toothache or discomfort due to your teeth during the past 12** | Often  Occasionally  Rarely  Never  Don’t know | 1  2  3  4  9 |
| **some aspects of self-assessed oral health problem** | Dissatisfaction with the appearance of my teeth  Avoiding smiling and laughing because of my teeth  Other children make fun of my teeth  Missing classes or miss school days due to Toothache or discomfort  Eating difficulty (in biting hard foods or in chewing) | 1 (Yes) /2 (No) |

All response options have been entered into the dataset. However, some response options have been combined during the analysis for a simpler, uncomplicated presentation of the results.

**References**

1.	Somaliland Map2021. [Available from: <https://ontheworldmap.com/somaliland/>]

2.	WHO. *Oral health surveys: basic methods*: World Health Organization; 2013. https://www.who.int/publications/i/item/9789241548649
